# Evidence for a genetic contribution to the ossification of spinal ligaments in Ossification of Posterior Longitudinal Ligament and Diffuse idiopathic skeletal hyperostosis: A narrative review

**DOI:** 10.3389/fgene.2022.987867

**Published:** 2022-10-07

**Authors:** Ana Rita Couto, Bruna Parreira, Deborah M. Power, Luís Pinheiro, João Madruga Dias, Irina Novofastovski, Iris Eshed, Piercarlo Sarzi-Puttini, Nicola Pappone, Fabiola Atzeni, Jorrit-Jan Verlaan, Jonneke Kuperus, Amir Bieber, Pasquale Ambrosino, David Kiefer, Muhammad Asim Khan, Reuven Mader, Xenofon Baraliakos, Jácome Bruges-Armas

**Affiliations:** ^1^ Hospital de Santo Espirito da Ilha Terceira EPER, SEEBMO, Angra do Heroísmo, Portugal; ^2^ Comprehensive Health Research Centre, Hospital de Santo Espírito da Ilha Terceira, Lisbon, Portugal; ^3^ University of Algarve, Center of Marine Science (CCMAR), Faro, Portugal; ^4^ Hospital de Santo Espirito da Ilha Terceira EPER, Orthopedics Service, Angra do Heroísmo, Portugal; ^5^ Centro Hospitalar Do Medio Tejo EPE Unidade de Torres Novas, Rheumatology Department, Santarém, Portugal; ^6^ CHRC Campus Nova Medical School, EpiDoc Research Unit, CEDOC, Lisboa, Portugal; ^7^ Emek Medical Center, Rheumatology Unit, Afula, Israel; ^8^ Sheba Medical Center, Tel Aviv, Israel; ^9^ Luigi Sacco University Hospital, Rheumatology Unit, Milano, Italy; ^10^ Istituti Clinici Scientifici Maugeri IRCCS, Neuromotor Rehabilitation Unit of Telese Terme Institute, Pavia, Italy; ^11^ Universita Degli Studi di Messina, Rheumatology Unit, Clinical and Experimental Medicine, Messina, Italy; ^12^ University Medical Centre, Department of Orthopedics, Utrecht, Netherlands; ^13^ Universitair Medisch Centrum Utrecht, Utrecht, Netherlands; ^14^ Istituti Clinici Scientifici Maugeri IRCCS, Cardiac Rehabilitation Unit of Telese Terme Institute, Pavia, Italy; ^15^ Ruhr-Universitat Bochum, Rheumazentrum Ruhrgebiet, Bochum, Germany; ^16^ Case Western Reserve University, Cleveland, OH, United States; ^17^ Rappaport Faculty of Medicine, Technion, Haifa, Israel; ^18^ Ruhr University Bochum, Rheumazentrum Ruhrgebiet, Herne, Germany

**Keywords:** ossification, genetics, ectopic calcification, diffuse idiopathic skeletal hyperostosis, ossification of posterior longitudinal ligament

## Abstract

Diffuse Idiopathic Skeletal Hyperostosis (DISH) and Ossification of the Posterior Longitudinal Ligament (OPLL) are common disorders characterized by the ossification of spinal ligaments. The cause for this ossification is currently unknown but a genetic contribution has been hypothesized. Over the last decade, many studies on the genetics of ectopic calcification disorders have been performed, mainly on OPLL. Most of these studies were based on linkage analysis and case control association studies. Animal models have provided some clues but so far, the involvement of the identified genes has not been confirmed in human cases. In the last few years, many common variants in several genes have been associated with OPLL. However, these associations have not been at definitive levels of significance and evidence of functional significance is generally modest. The current evidence suggests a multifactorial aetiopathogenesis for DISH and OPLL with a subset of cases showing a stronger genetic component.

## 1 Introduction

The spine is a columnar structure composed of bony vertebrae interconnected by intervertebral discs and supported by ligaments, such as the anterior and posterior longitudinal ligaments, ligament nuchae and ligamentum flavum. The spinal canal, enclosed within the foramen of the vertebrae, contains the spinal cord. In the intervertebral spaces, the canal is protected by the ligament flavum posteriorly and the posterior longitudinal ligament anteriorly. Spinal stenosis consists in the reduction of the area of the spinal canal, leading to motor neuron deficits and related neurological symptoms, depending on the location of the stenosis (Bai et al., 2022). In the elderly population, the most common cause of spinal cord impairment is the degenerative cervical myelopathy (DCM). DCM can be secondary to osteoarthritic degeneration or to ligamentous ossifications such as the Ossification of the Posterior Longitudinal Ligament (OPLL) or the ossification of the Ligament Flavum (OLF) (Nouri et al., 2015). OPLL, frequently in association with DISH, can result in various degrees of neurological complications that can range from a slowly progressive painless myelopathy to a rapid progression of a neurological deficit even after minor injury (Takayuki et al., 2021; Prabhu et al., 2022). The physical and socioeconomic burden of disability associated with DCM is expected to grow evenly, due to the ageing population (Badhiwala et al., 2020). It is thus crucial to improve the diagnosis and assessment of disorders involved in DCM for early detection and swift intervention.

This review will focus on genetic studies of the ossification of the anterior and posterior longitudinal ligaments, the Diffuse Idiopathic Skeletal Hyperostosis (DISH) [MIM: 106400] and the Ossification of the Posterior Longitudinal Ligament (OPLL) [MIM: 602475], respectively. A short outline of DISH, OPLL and OLF can be seen in Table 1. These conditions may co-occur in some patients suggesting possible common etiopathogenic factors (Nouri et al., 2015; Takayuki et al., 2021). The objective was to collect and present evidences that supports a genetic foundation, based on the following observations: 1) familial aggregation reports, 2) animal models, 3) associated genetic variants and 4) genetics of associated disorders.

**TABLE 1:** Brief characterization, main symptoms and epidemiology of DISH, OPLL and OLF.

Disorder	Characterized by	Main symptom	Epidemiology
DISH	Calcification and ossification of the anterior longitudinal ligament affecting, in particular, the right side of the spine with preservation of the intervertebral disc space. Peripheral joints, such as elbow, shoulder, hip, knee and heel are commonly affected (Okazaki et al., 1976; Gorman et al., 2005; Bruges-Armas et al., 2006; Couto et al., 2017; Parreira et al., 2020)	Dysphagia (Beardwell, 1969). Axial pain, elbow, knee and metacarpophalangeal pain, swelling and deformity (Okazaki et al., 1976)	Elderly males are mostly affected. DISH prevalence is 17.6% using x-ray and ranges from 17.4% to 27,2% using computed tomography [33, (Ikuma et al., 2022)
OPLL	Ectopic hyperostosis and calcification of the posterior longitudinal ligament at the cervical, thoracic and lumbar spine (Fornaciari et al., 2009)	Myelopathy and/or radiculopathy (Fornaciari and Giuffra, 2013)	More common in males of asian populations, with a prevalence of 2–4% in japan as compared with 0.01–2% in non-Asian populations (Matsunaga et al., 2006)
OLF	Calcification of the ligamentum flavum (LF) not extending to the closed spinal bony arch (Yamagami et al., 2000). Calcium pyrophosphate dehydrate (CPPD) and hidroxyapatite are thought to be main players in this calcification (Ellman et al., 1978; Brown et al., 1991)	Thoracic myelopathy and spinal stenosis (Miyasaka et al., 1982)	Higher prevalence in males of Asian populations, especially the Japanese, with the incidence of 12% in thoracic OLF (Caswell et al., 1987)

## 2 Familial aggregation reports

### 2.1 DISH

Reports of familial DISH are scarce. Beardwell, A. in 1969 (Beardwell, 1969), describes a family with Ankylosing Vertebral Hyperostosis (AVH), by the third decade, with many family members also presenting tylosis (punctuate hyperkeratosis). As demonstrated by the author, the X-ray of the affected family members showed ossification of paraspinal distribution, mainly in the lower thoracic region and also some osteophytosis and marginal sclerosis of the sacroiliac joints.

Another report of familial DISH, described 2 families; one had 4 siblings showing AVH by the fourth decade and two other family members had probably AVH. The second family was dentified after hip surgery of two sisters aged 71 and 82 years. The proband had five daughters, two of them affected by AVH and other two with a mild phenotype, classified as possible AVH (Abiteboul et al., 1985). An unusual DISH-like phenotype was described in a family with severe cervical disease lacking the extensive dorsal involvement (Gorman et al., 2005).

In Azores region, twelve families were identified presenting early onset (third decade) of DISH and/or Chondrocalcinosis (CC). The affected members had a pyrophosphate arthropathy showing exuberant axial and peripheral enthesopathic calcifications, meaning calcification of the connective tissues in the attachments of tendons or ligaments to the bones, in joints other than the spine (Bruges-Armas et al., 2006). Genetic studies in these families suggest that the phenotype DISH/CC is polygenic and influenced by the interaction of several, small-effect gene variants and possibly by unidentified environmental factors (Couto et al., 2017; Parreira et al., 2020). Similar cases, of patients with CPPD and/or CC and DISH, were mentioned in other studies (Okazaki et al., 1976), also showing familial aggregation (van der Korst et al., 1974; Bruges-Armas et al., 2006).

A postmortem examination of a skeleton allowed the diagnosis of DISH and ankylosing spondylitis in the same patient (Jordana et al., 2009). An extensive radiographic survey on several members of the Medici family (15th–17th century), demonstrated that DISH, rheumatoid arthritis and uric acid gout affected several family members (Fornaciari et al., 2009; Fornaciari and Giuffra, 2013). A study of 13 royal Egyptian mummies detected ossifications at the anterior aspects of the spines in five male mummies but only four fulfilled the criteria for DISH (Saleem and Hawass, 2014).

### 2.2 OPLL

The cause of OPLL is unclear but people of Asian heritage, have a higher likelihood of developing this condition (Choi et al., 2011). Familial aggregation of cervical OPLL was first demonstrated in a study assessing 347 families (Terayama, 1989); the relative risk of first degree relatives came to have OPLL was five times greater than expected in the general population. Another study shows a prevalence of 27% with a relative risk seven times that of the general population (Tanikawa et al., 1986). Other OPLL familial cases included the report of familial thoracic OPLL in Caucasian siblings (Tanabe et al., 2002) (Terayama, 1989).

The mode of inheritance for OPLL is still poorly defined due to the absence of large families, late onset of the disorder, environmental effects and sex differences (Koga et al., 1998). However, segregation studies shows that OPLL have both autosomal dominant (Tanikawa et al., 1986) and autosomal recessive (Hamanishi et al., 1995) patterns of inheritance. As discussed later, ectopic ossification resembling OPLL, as seen in the tiptoe walking mouse (ttw) or also called tiptoe walking of Yoshimura (twy), is inherited as an autosomal recessive disease with complete penetrance (Ikegawa et al., 2007).

## 3 Animal models for Ossification of Spinal Ligaments

The study of mouse strain models and the progress of strategies to find genetic mutations, affecting the mineralization pattern, have permitted the discovery of many genes and proteins to be evaluated.

### 3.1 DISH

**Table:** 

Natural cases—unknown gene
Some natural cases of DISH have been observed in dogs (Kranenburg et al., 2010; Kranenburg et al., 2014; Togni et al., 2014; Bossens et al., 2016) and, as in humans, the disease is more common in older male animals and is more frequent in the boxer breed (Kranenburg et al., 2010). The high occurrence of DISH in one dog breed and the low or absence occurrence in the other breeds is suggestive of a genetic mechanism (Ostrander et al., 2000). In 2016 Bossens et al. (2016), reported the presence of DISH, in a nine year old female cat. According to the authors the phenotype was very similar to canine DISH displayed contiguous ossification ventral and lateral to the vertebra prolonging from thoracic area to the lumbosacral junction. As far as we know, there are no reports of OPLL in dogs or other types of companion animals
Gene involved in humans—*ENT1*
***ENT1*** (6p21.1) in humans is known as solute carrier family 29 member 1 (***SLC29A1***). The gene encodes one of the four equilibrative nucleoside transporters which transfers hydrophilic nucleosides across the plasma membrane (Bicket et al., 2016). The protein is ubiquitously expressed and is involved in purine metabolism being responsible for transporting the majority of adenosine. It is known that adenosine signaling regulate bone formation (Carroll et al., 2012). Currently no human phenotype or disease has been directly linked with this gene
*ENT1* ^*−/−*^ *mice*
Mice lacking *ENT1* (*ENT1* ^***−/−***^) exhibit progressive ectopic calcification of the paraspinal tissues in the cervical and thoracic area homologous to human DISH. In intervertebral discs, these mice also present a significant downregulation of *Enpp1*, *Ank* and *Alpl* genes (Warraich et al., 2013). Another study, showed that *ENT1−/−* mice presented low bone density in the midshaft of the femur and in the lower half of the spinal column. Additionally, the authors confirmed that ***ENT1*** ^***−/−***^ mice presented osteoid formations in the thoracic and cervical portions of the spinal column (Hinton et al., 2014)

### 3.2 OPLL

**Table:** 

Gene involved—*ENPP1*
In humans, *ENPP1* (6q23.2) encodes one of the seven members of the ectonucleotide pyrophosphate phosphodiesterase family (Buckley et al., 1990). ENPP1 is a membrane glycoprotein responsible to hydrolysing extracellular nucleotide triphosphates (ATP) to generate pyrophosphate, thereby working as a physiological inhibitor of calcification (Stefan et al., 2005) (Kato et al., 2012). The protein is expressed in various tissues, including bone and cartilage (Caswell et al., 1987; Caswell and Russell, 1988). Some human diseases are linked to this gene. It is known that mutations in *ENPP1* gene are the cause of Generalized arterial Calcification of Infancy (GACI) (Rutsch et al., 2003), Hypophosphatemic rickets (Levy-Litan et al., 2010), Cole disease (Eytan et al., 2013) and Pseudoxanthoma elasticum, since in some GACI cases, mutations in *ENPP1* also caused a characteristic pseudoxanthoma skin lesions and angioid streaks of the retina (Nitschke and Rutsch, 2012)
*Twy walking Yoshimura mouse*
The spinal hyperostotic mouse twy develop spontaneous ossification of the spinal ligaments very similar to human OPLL. The ossification also occurs in joint capsules, chondral tissues, tendon entheses and peripheral ligaments (Yamazaki et al., 1991) (Okawa et al., 1998). The twy phenotype is caused by a nonsense mutation in *NPPS* also called *ENPP1* gene, resulting in a minor expression and consequently less protein activity (Okawa et al., 1998). According to Hajjawi et al. (2014), *ENPP1* ^*−/−*^ knock-out mice also shown a lower bone density and calcification of joints, vertebrae and soft tissues including trachea, ear pinna and whisker follicles.This mouse model has also been used for studies on the contribution of Fas-mediated cell death and inflammation to the pathobiology of cervical spondylotic myelopathy (Yu et al., 2011)
Gene involved - *LEP/LEPR*
In humans, *LEP* (7q32.1) encodes a protein responsible to regulate energy homeostasis. The protein is related to bone metabolism since is a potent inhibitor of bone *in vivo* (Elefteriou et al., 2004). In female mice, the protein promotes the transdifferentiation of vascular smooth muscle cells to osteoblasts by increasing RANKL expression (Liu et al., 2014) In humans, mutations in *LEP* gene cause morbid obesity (Montague et al., 1997)
ZFR rat
The Zucker fatty rat (ZFR) was originally used to study obesity, hyperinsulinemia, hypercholesterolemia and hyperlipidemia. This murine model also displays ossification of the spinal ligaments, histopathologically similar to human OPLL (Okano et al., 1997). The ZFR phenotype is caused by a mutation in the leptin receptor gene (*LEPR*) (Phillips et al., 1996)

## 4 Genetic variants associated with OSL in humans

### 4.1 Genetic studies of DISH

Some of the earliest genetic studies were performed on genes belonging to Major Histocompatibility complex, specifically Human Leucocyte Antigens (HLA) (Brewerton et al., 1973; Schlosstein et al., 1973), but this association was never confirmed.

In a small study, polymorphisms of the Collagen Type I Iα1 (COL1A1), and Vitamin D Receptor (VDR) were investigated, but the authors concluded that these genes do not seem to be related to DISH etiology (Havelka et al., 2002). One more study, investigated polymorphisms of the collagen 6A1 gene (*COL6A1*) in Czech and Japanese DISH patients and the polymorphism, in intron 32, was associated with the disorder in Japanese patients but failed the association test with DISH Czech patients (Table 2). However, the authors suggested that *COL6A1* could be related to ectopic bone formation in spinal ligaments (Tsukahara et al., 2005). Due to the possible common aetiopathogenesis of OPLL and DISH, a genotyping study (intron 6; −4) on the *COL11A2* gene was performed, and no significant difference was observed between both cohorts (Havelka et al., 2001). Jun et al (Jun and Kim, 2012) described that two polymorphisms in the *FGF2* gene were associated with DISH (Table 2). Another study identified a genetic variant in the *PPP2R2D* gene significantly associated with a phenotype characterized by DISH and CC. It was proposed that *PPP2R2D* may contribute to the development of this disorder (Parreira et al., 2020). Although these variants are significantly associated with DISH, the direct evidence for pathogenicity is lacking.

**TABLE 2:** Genes and genetic variants associated with DISH. The protein physiological function is also mentioned. Gene function was obtained from GeneCards database.

Gene	Chr	Gene function	Type of study	SNVs	Molecular mechanism	Ref
*COL6A1*	21	Collagen VI is a main structural component of microfibrils. Mutations in this gene may result in Bethlem Myopathy	Case control association study in Japanese individuals (97 DISH patients and 298 controls)	rs2236486 (*p* = 0.0022)	Frequent polymorphism (MAF 0.39)	Tsukahara et al. (2005)
					Unclear association	
*FGF2*	4	FGF2 protein has been involved in diverse biological processes, such as limb and nervous system development, tumour growing and wound healing	Case control association study (154 OPLL patients -3 patients with DISH)	rs1476217 (*p* = 0.003)	3 prime UTR variant (MAF 0.48)	Jun and Kim, (2012)
				rs3747676 (*p* = 0.002)	3 prime UTR variant (MAF 0.35)Unclear association	
*PPP2R2D*	10	PPP2R2D protein is a crucial serine/threonine protein phosphatase that controls basal cellular activities by dephosphorylating substrates. Its is known that phosphatases	Whole exome sequencing (4 patients) and case control study (n = 65)	rs34473884 (*p* = 0.028)	Missense variant (MAF 0.18)	Parreira et al. (2020)
		influence the transforming growth factor beta (TGF-beta) superfamily signalling, which regulates numerous cellular responses			Unclear association	

### 4.2 Genetic studies in OPLL

Many genetic studies of OPLL have been performed and it is now well established that genetic factors are implicated in its etiology (Terayama, 1989) (Table 3). In the same way as DISH, the initial genetic studies of OPLL were performed on HLA and the possible association is much discussed in the literature (Sakou et al., 1991; Yamaguchi, 1991; Matsunaga et al., 1999). Very close to the HLA region on the chromosome 6 is *COL11A2* and common variants of this gene have been associated with OPLL (Koga et al., 1998; Maeda et al., 2001a). The polymorphism in intron 6 (-4A) seems to confer protection to OPLL furthermore, it was proven that this polymorphism of *COL11A2* affects the splicing of exon 6 in cells obtained from spinal ligaments from OPLL patients (Maeda et al., 2001b).

**TABLE 3:** Genes and genetic variants associated with OPLL predisposition. The protein physiological function is also mentioned. Protein function was obtained from GeneCards database.

Gene	Chr	Physiological function	Study type	SNP ID - significantly associated	Association explained?	References
*IL-1β*	2	Stimulates thymocyte proliferation by promoting the IL-2 release, B-cell maturation and proliferation and fibroblast growth factor activity	Case-control association study with 120 OPLL (43 Female) patients and 306 controls (140 Female) (unrelated Japanese)	*IL1B AbaI* variant (gender specific—female) (*p* = 0.001)	Intronic polymorphism	Ogata et al. (2002)
			Assessed 5 candidate gene polymorphisms		Unclear association	
*AHSG*	3	Promotes endocytosis, possesses opsonic properties and influences the mineral phase of bone. AHSG protein have affinity for barium ions and calcium	Large Scale Case-control study in Japanese individuals. 711 OPLL patients and 896 controls	rs2077119 (*p* = 0.0011)	SNP in Promoter region	Horikoshi et al. (2006)
			Assessed 35 candidate genes; 109 SNPs		MAF 0.36 Unclear association	
*ACE*	17	Angiotensin converting enzyme-2 is important in the renin-angiotensin system	Case control association study in Korean individuals. 95 OPLL patients and 274 controls	rs4646994 (genotype DD *p* < 0.001; D allele *p* = 0.009)	SNP in intronic region	Kim et al. (2014b)
			Assessed I/D polymorphism in *ACE*		Unclear association	
*BMP2*	20	Induces bone and cartilage formation; member of TGFβ superfamily	Case control study with 192 OPLL patients and 304 controls	rs3178250 (*p* = 0.003 gender specific—males)	3 prime UTR variant	Wang et al. (2008)
			Assessed 2 SNPs in Exon 3 of BMP2		MAF 0.27 Unclear association	
			Case control study with 57 OPLL patients and 135 controls	rs2273073 (*p* <0.001) susceptibility to OPLL	Missense variant	Chen et al. (2008)
			Assessed 2 SNPs in exon 2 of *BMP2* gene	rs1049007 (p=<0.001) severity of OPLL	MAF 0.03 Synonymous variant MAF 0.25 Unclear association	
			Case control study with 420 OPLL patients and 506 controls	rs2273073 (*p* < 0.001)	Missense mutation (MAF 0.03)	Yan et al. (2013)
			Assessed all coding sequencing of *BMP2* gene	rs235768 (*p* = 0.005)	Missense—Deleterious (MAF 0.23) This study provides evidence that the mutation (rs2273073) is associated with level of Smad4 protein expression and activity of ALP.	
*BMP4*	14	BMP4 protein promotes bone and cartilage formation	Nonparametric linkage study with 126 affected sib-pairs	Only *BMP4* gene reached criteria of suggestive evidence of linkage (NPL = 2.23; *p* = 0.035)	Molecular variants not identified	Furushima et al. (2002)
			Used microsatellite markers in 88 candidate genes		Unclear association	
			Case control association study in Chinese individuals. 179 OPLL patients and 298 controls	rs17563 (genotype: *p* = 0.039; Allele: *p* = 0.014)	Missense variant	Meng et al. (2010)
			Assessed 2 polymorphisms in *BMP4* gene		MAF 0.33Unclear Association	
			Association study in Chinese individuals. 450 OPLL patients and 550 matched controls	rs17563	Missense variant	Ren et al. (2012a)
				rs76335800	MAF 0.33 (benign)	
					3 prime UTR variant MAF 0.30 (Benign)	
			Complete genomic *BMP4* coding		Unclear association	
*BMP9*	10	BMP9 has been called as a osteogenic, and chondrogenic factor and it could be involved in bone formation	Association study in Chinese individuals. 450 OPLL patients and 550 matched controls	rs75024165 (*p* < 0.001)	Missense variant	Ren et al. (2012b)
			Complete genomic *BMP9* coding	rs34379100 (*p* < 0.001)	MAF <0.01 (Benign)	
					3 prime UTR variant MAF 0.17 (3′ Region) Unclear association	
*COL11A2*	6	COL11A2 protein may promote ectopic bone formation by enhancing endochondral ossification. In addition COL11A2 also play a role in fibrillogenesis	Genetic linkage, association and haplotype analysis study in 53 Japanese families containing 91 OPLL affected sib pairs	Promoter (−182) (*p* = 0.02)	Linkage, association and haplotype analysis suggestive of a genetic locus for OPLL susceptibility in chromosome 6p, within or near COL11A2	Koga et al. (1998)
				rs1799907 (*p* = 0.0004)		
				rs1799910 (*p* = 0.02)		
				rs1799911 (*p* = 0.03)		
				Haplotypes		
			Association study (Haplotype association) in 161 OPLL patients and 163 controls	rs1799907 (*p* = 0.0003)	This study provides evidence of the functional impact of rs1799907 as a splice site mutation (MAF 0.32) which confers protection against ossification	Maeda et al. (2001a)
				Haplotype with 4 SNPs, male association		
*COL17A1*	10	COL17A1 is involved in the integrity of the hemidesmosome and the attachment of basal keratinocytes to the underlying basement membrane	WES and association studies in Chinese individuals. 28 unrelated OPLL patients and 100 healthy controls	rs805698 (*p* = 0.00023)	Missense variant (MAF 0.18) Tolerated effect	Wei et al. (2014)
				rs4918079 (*p* = 0.003)	Synonymous variant (MAF 0.33) Unclear association	
*COL6A1*	21	COL6A1 is a cell binding protein involved in the increase of bone mass	Genomewide linkage study followed by fine mapping and haplotype analysis of 142 affected sib pairs. 280 OPLL patients and 210 controls	intron 32 (−29) (*p* = 0.000003)	Identified COL6A1 as strongly associated with OPLL but did not find any functional impact of the identified polymorphisms	(Tanaka et al., 2003; Kong et al., 2007)
				rs2236485 (*p* = 0.0002) (MAF 0.13)		
				rs2236486 (*p* = 0.00005) (MAF 0.39)		
				rs2236487 (*p* = 0.00006) (MAF 0.37)		
			Case control association study with 90 OPLL patients and 155 controls	Promoter (−572) (*p* = 0.000215)	Promoter variant	Kong et al. (2007)
				Intron 32 (−29) rs2236486 (*p* = 0.00483)	Frequent Intronic variant - MAF 0.39 Unclear association	
			Association study with 100 OPLL patients and 100 controls (Han Chinese). Assessed 3 SNPs, previous identified by whole genome sequencing, in 30 OPLL patients (Wang et al., 2018b)	rs201153092 (*p* = 0.000114)	Missense, MAF<0.01	Wang et al. (2018a)
				rs13051496 (*p* = 0.01116)	Missense, MAF 0.11	
*ENPP1*	6	ENPP1 play a key role in bone mineralization and soft tissue calcification by controling pyrophosphate levels	ttw mouse studies	Gly568stop	Mouse Model for OPLL with nonsense mutation originating a truncated protein with loss of enzymatic activity	Okawa et al. (1998)
			Association study using 323 OPLL patients and 332 controls	IVS20-11delT (*p* = 0.0029)	Frequent polymorphism	Nakamura et al. (1999)
			Assessed all coding sequencing of ENPP1 gene		Unknow pathological mechanism of association with disease	
			Case-control association study with 180 OPLL patients and 265 controls	IVS15-14T-- > C (*p* < 0.0001)	Highly Significate in Young female and severe OPLL patients. Unknown pathological mechanism of association with disease	Koshizuka et al. (2002)
			Association study with 95 OPLL Chinese patients and 90 controls. Assessed 4 SNPs in ENPP1	C973T (*p* < 0.001)	Unclear association	He et al. (2013)
				IVS15-14T-C (*p* = 0.026)		
*ESR1*	6	ESR1 protein play a role in bone tissues and is essential for sexual development and reproductive function	Case-control association study with 120 OPLL patients (43Female) and 306 controls (140Female)—unrelated Japanese	ER (XbaI) female gender specific	Intronic polymorphism	Ogata et al. (2002)
			Assessed 5 genes; 5 SNPs	(*p* = 0.007)	Unclear association	
			Large Scale Case-control study of 711 Japanese OPLL patients and 896 controls	rs9340799 (*p* = 0.017), no correction	Frequent Intronic polymorphism	Horikoshi et al. (2006)
			Assessed 35 candidate genes; 109 SNPs	rs2228480 (*p* = 0.034, no correction	Unclear association	
*HLA*	6	HLA is closely related in the presentation of foreign antigens to the immune system	Family based association study in 33 families of patients with OPLL.		Unclear Association	Sakou et al. (1991)
			Family based association study in 24 families of patients with OPLL.		Unclear Association	Matsunaga et al. (1999)
*IL-15RA*	10	Increase cell proliferation and expression of an apoptosis inhibitor	A case control study in Chinese individuals. 235 OPLL patients and 250 controls	rs2228059	Tolerated missense variant MAF 0.45	Guo et al. (2014)
					Unclear association	
			Association study in Korean individuals. 166 OPLL patients and 230 controls	rs2228059	Tolerated missense variant MAF 0.45	Kim et al. (2011)
					Unclear association	
*IL-17RC*	3	IL-17RC is involved in regulation of bone metabolism by accelerating osteoblast differentiation	Association study in Han Chinese individuals. 100 OPLL patients and 100 controls	rs199772854 (*p* = 0.006515) rs76999397 (*p* = 0.003234) rs189013166 (*p* = 0.01827)	Missense variant MAF<0.01 Synonymous variant MAF 0.03 Synonymous variant MAF 0.02	Wang et al. (2018a)
			Assessed 3 SNPs, previously identified by whole genome sequencing, in 30 OPLL patients (Wang et al., 2018b)			
*RUNX2*	6	RUNX2 play a role in osteoblastic differentiation and skeletal morphogenesis	Case control study (Sequenom system) in Chinese individuals. 82 OPLL patients and 118 controls	rs1321075 (*p* = 0.043)	Intron Variant	Liu et al. (2010)
			Assessed 19 SNPs in 4 candidate genes	rs12333172 (*p* = 0.034)	MAF 0.18 Intronic variant MAF 0.13 Unclear association	
			Association study with 80 OPLL patients and 80 controls	rs1321075	Intron variant, MAF 0.18 Intron variant, MAF 0.13 Intron variant, MAF 0.47	Chang et al. (2017)
			Assessed 3 SNPs	rs12333172 rs1406846		
*RXRB*	6	RXRB protein is a member of retinoid receptor family, involved in regulation of a wide variety of biological processes including development, differentiation, and cellular metabolism	Association study and haplotype analysis in Japanese individuals. 134 OPLL patients and 158 controls	3′UTR (+140) (*p* = 0.0028)	Unclear association	Numasawa et al. (1999)
				3′UTR (+561) (*p* = 0.034)		
*TGFB1*	19	TGFB1 mediates bone development and metabolism	A case control with 46 OPLL patients and 273 controls	rs1982073 (p =)	Frequent Polymorphism	Kamiya et al. (2001)
					MAF 0.45	
					Unclear association	
*TGFB3*	14	Involved in embryogenesis and cell differentiation	Large Scale Case-control study in Japanese individuals. 711 OPLL patients and 896 controls	rs2268624 (*p* = 0.00040/*p* = 0.044 after Bonferoni Correction)	Intronic polymorphisms with high MAFs	Horikoshi et al. (2006)
			Assessed 35 candidate genes; 109 SNPs	rs2284792 (*p* = 0.037) no correction	Unclear association	
*VDR*	12	Plays a central role in calcium homeostasis	Case-control study with 63 OPLL patients and 126 controls	VDR FF genotype	Unclear association	Kobashi et al. (2008)
*RSPH9*	6	Plays a role in membranous ossification	Genome Wide association study in Japanese individuals. 1130 OPLL patients and 7135 controls followed by an association study (for replication) in 548 OPLL Japanese patients and 6469 controls	rs927485 (*p* = 9.4 × 10–^9^)	Trough Gene expression analysis in and around OPLL associated loci authors suggest that *RSPH9* and *STK38L* genes might be linked in OPLL aetiology through the membranous ossification process. Furthermore, *HAO1*, *RSPO2* and *CCDC91* genes might be involved through the endochondral ossification process	(Nakajima et al., 2014; Nakajima et al., 2016)
*STK38L*	12	Plays a role in the membranous ossification process		rs11045000 (*p* = 2.95 × 10–^11^)		
*RSPO2*	8	Implicated in the endochondral ossification process		rs374810 (*p* = 1.88 × 10–^13^)		
				rs13279799 (*p* = 1.28 × 10–^10^)		
*CCDC91*	12			rs1979679 (*p* = 4.34 × 10–^12^)		
*HAO1*	20	Implicated in the endochondral ossification process		rs2423294 (*p* = 1.10 × 10–^13^)		
*FGFR1*	8	Plays an essential role in the regulation of embryonic development, cell proliferation, differentiation and migration	Association study with 157 OPLL patients and 222 controls	rs13317 (*p* = 0.02)	3 prime UTR variant	Jun and Kim, (2012)
			Assessed 9 SNPs in 3 genes		MAF 0.23 Unclear Association	
*BID*	22	Has a role in apoptosis signaling	Association study with 157 Korean OPLL patients and 209 controls	rs8190315 (*p* = 0.0052)	Tolerated Missense Variant MAF 0.05 Synonymous variant	Chon et al. (2014)
			Assessed 2 coding SNPs in BID	rs2072392 (*p* = 0.0052)	MAF 0.05 Unclear association	
*TGFBR2*	3	TGFBR2 protein is a regulator of transcription of several genes related to cell proliferation	Association study with 21 OPLL patients and 42 controls	rs11466512 (*p* = 0.007)	Splice region variant MAF 0.27 Rare Benign Missense Variant MAF <0.01 Unclear Association	Jekarl et al. (2013)
				rs56105708 (*p* = 0.024)		
*VKORC1*	16	Involved in vitamin K metabolism	Association study with 98 Korean OPLL patients and 200 controls	rs9923231 (*p* = 0.004) (female)	Uppstream gene variant MAF 0.36	Chin et al. (2013)
					Unclear Association	
*IFNG*	12	IFNG is a protein that activates the macrophages	Association study with 135 OPLL patients and 222 controls	rs2430561	Intronic Variant MAF 0.28 Tandem repeat Unclear association	Kim et al. (2012)
				rs3138557		
*BMPR-IA*	10	Bone morphogenetic protein receptor responsible for the initiation of osteogenic differentiation	Association study with 356 OPLL patients and 617 controls. (Han Chinese)	rs11528010 (4A < C) (*p* < 0.001)	Missense variant MAF 0.50 5′UTR MAF 0.35	Wang et al. (2018c)
			Assessed all exon regions of *BMPR-IA* gene	rs34755052 (-349C > T) (*p* < 0.001)		
*MiR-199*	19	Involved in regulation of inflammation and chondrogenic differentiation	Association study in Korean individuals. 207 OPLL patients and 200 controls	rs3746444 (*p* = 0.039)	Non-coding transcript exon variant	Lim et al. (2016)
			Assessed 4 genes/SNPs		MAF 0.18	

Abbreviations: *IL-1β*: Interleukin 1 Beta, *AHSG*: Alpha 2-Heremans-Schmid glycoprotein, *ACE*: Angiotensin I Converting Enzyme, *BMP2*: Bone Morphogenetic Protein 2, *BMP4*: Bone Morphogenetic Protein 4, *BMP9*: Bone Morphogenetic Protein 9, *COL11A2*: Collagen Type XI, Alpha 2, *COL17A1*: Collagen Type XVII, Alpha 1, *COL6A1*: Collagen Type VI, Alpha 1, *ENPP1*: Ectonucleotide pyrophosphatase/phosphodiesterase 1, *ESR1*: Estrogen Receptor 1, *HLA*: Human Leukocyte antigen, *IL-15RA*: Interleukin 15 Receptor Alpha, *IL-17RC*: Interleukin-17, receptor C, *RUNX2*: Run-Related Transcription Factor 2, *RXRB*: Retinoid X Receptor Beta, *TGFβ1*: Transforming Growth factor Beta 1, *TGFβ3*: Transforming Growth factor Beta 3, *VDR*: Vitamin D Receptor, *RSPH9*: radial spoke head 9 homolog, *STK38L*: serine/threonine kinase 38 like, *RSPO2*: R-spontin 2, *CCDC91*: Coiled-coil domain containing 91, *HOA1*: Hydroxyacid oxidase 1, *FGFR1*: Fibroblast Growth Factor Receptor 1, *BID*: BH3 Interacting Domain Death Agonist, *TGFBR2*: Transforming Growth Factor Beta Receptor II, *VKORC1*: Vitamin K epoxide reductase complex subunit 1, *IFNG*: Interferon Gamma, *BMPR-IA*: Bone morphogenetic protein receptor type IA. NA: Not applicable. * This polymorphisms was found in all OPLL, patients and according to the authors is a novel nucleotide variation.

According to Nakamura et al. (1999) the deletion of T, 11 nucleotides upstream of the splice acceptor site of intron 20 (IVS20-11delT) of *ENPP1* is associated with OPLL. However, He et al. (2013) described that the polymorphism TT genotype of C973T and IVS15-14T as well as the wild type IVS20 (lack of deletion) were related with disease severity. Another study found a polymorphism (IVS15-14T-- > C) in *ENPP1* gene associated with OPLL susceptibility and severity (Koshizuka et al., 2002). Interestingly, in one study the authors found that the ENPP1 variant (IVS20-11delT) and the SNP (A861G) in the leptin receptor gene (LEPR) were more frequent in OPLL patients affected in the thoracic spine compared to patients whose OPLL was restricted to cervical spine. The authors suggested that the two variants (IVS20-11delT and A861G) are associated with more extensive OPLL, but not with frequency of its occurrence (Tahara et al., 2005).

The *COL6A1* gene is intensely associated to OPLL and polymorphisms in this gene are considered useful markers of OPLL (Tanaka et al., 2003; Kong et al., 2007; Wang et al., 2018a). However this association is not always confirmed in all the studies performed (Furushima et al., 2002; Liu et al., 2010). Polymorphisms in *COL6A1* gene were associated with DISH in the Japanese population (Tsukahara et al., 2005) suggesting that *COL6A1* may contribute in pathological ectopic ossification.

Positive associations of *BMP2,* an important regulator of bone metabolism, with OPLL were found with the SNPs rs3178250 (Wang et al., 2008), rs2273073 (Chen et al., 2008; Yan et al., 2013) and rs1949007 (Chen et al., 2008) (Table 3). Yan et al. (2013), confirmed that the SNP rs227373 in the *BMP2* gene is associated with the higher level of Smad4 protein expression and with activity of alkaline phosphatase. On the other hand, according to Kim et al. (2014a) the SNPs rs2273073 and rs1949007, in Korean patients, are not associated with OPLL. Other study (Liu et al., 2010), performed in Chinese Han population, also failed to show association between *BMP2* gene and OPLL. A genome-wide linkage study performed with 214 OPLL affected sib-pairs identified a chromosome region (20p12), linked with OPLL (Karasugi et al., 2013). This region contains 25 genes, of which two are good candidates: Jagged 1 (*JAG1*), which is involved in endochondral bone formation (Nobta et al., 2005) and *BMP2.* Furthermore, deleterious coding variants of *BMP2* in peripheral blood samples was recently demonstrated (Chen et al., 2016). Three other polymorphisms (rs996544, rs965291 and rs1116867) were screened in Han Chinese subjects and the authors found that rs1116867 and rs965291 were related with the manifestation and extend of OPLL (Yan et al., 2010).

Other bone morphogenetic protein genes have been associated with OPLL; two SNPs in *BMP-9* were found to be associated with OPLL: rs75024165 and rs34379100 (Ikuma et al., 2022). *BMP-4* SNPs rs17563 (Mader et al., 2013; Cudrici et al., 2021), rs76335800 and a specific haplotype, TGGGCTT (Mader et al., 2013), were identified as risk factors for developing OPLL in the Chinese population. Furushima et al. (Ramos et al., 2015) also confirmed the association of *BMP-4* with OPLL, in a large scale screening study, in which only *BMP-4* reached criteria of suggestive evidence of linkage. In a recent study, BMP-4 has even been proposed as a new therapeutic option for treating bone diseases due to its role on a RUNX2/CHRDLI/BMP4 pathway. Several SNPs in gene *RUNX2* have also been associated with OPLL (Liu et al., 2010; Chang et al., 2017).

Another important gene with contradictory results is *TGFβ1,* that *a*ccording to Kamiya et al. (2001), is genetically associated to OPLL (869T > C; rs1982073). However, Han et al. (2013) showed that the SNP previously associated with OPLL (rs1982073) and the SNP located in the promoter region (rs1800469) are not associated with OPLL in Korean populations. Interestingly, in the chondrocytes of adjacent cartilaginous areas and in the ossified matrix of OPLL the TGF-β1 gene is overexpressed. The same authors tested the association between rs1982073 and the radiological appearance of OPLL, and they verified that SNP rs1982073 is associated to the specific area of the ossified lesion, and not to the onset of OPLL. The “C” allele could be a risk factor for patients with OPLL in cervical, thoracic, and/or lumbar spine (Kawaguchi et al., 2003).

In relation to ossification of the ligamentum flavum several genes and loci have been associated with thoracic Ossification of Ligamentum Flavum (OLF) (Kong et al., 2007; Liu et al., 2010; Qu et al., 2017; Qu et al., 2021).

## 5 Associated disorders

**FIGURE 1:**
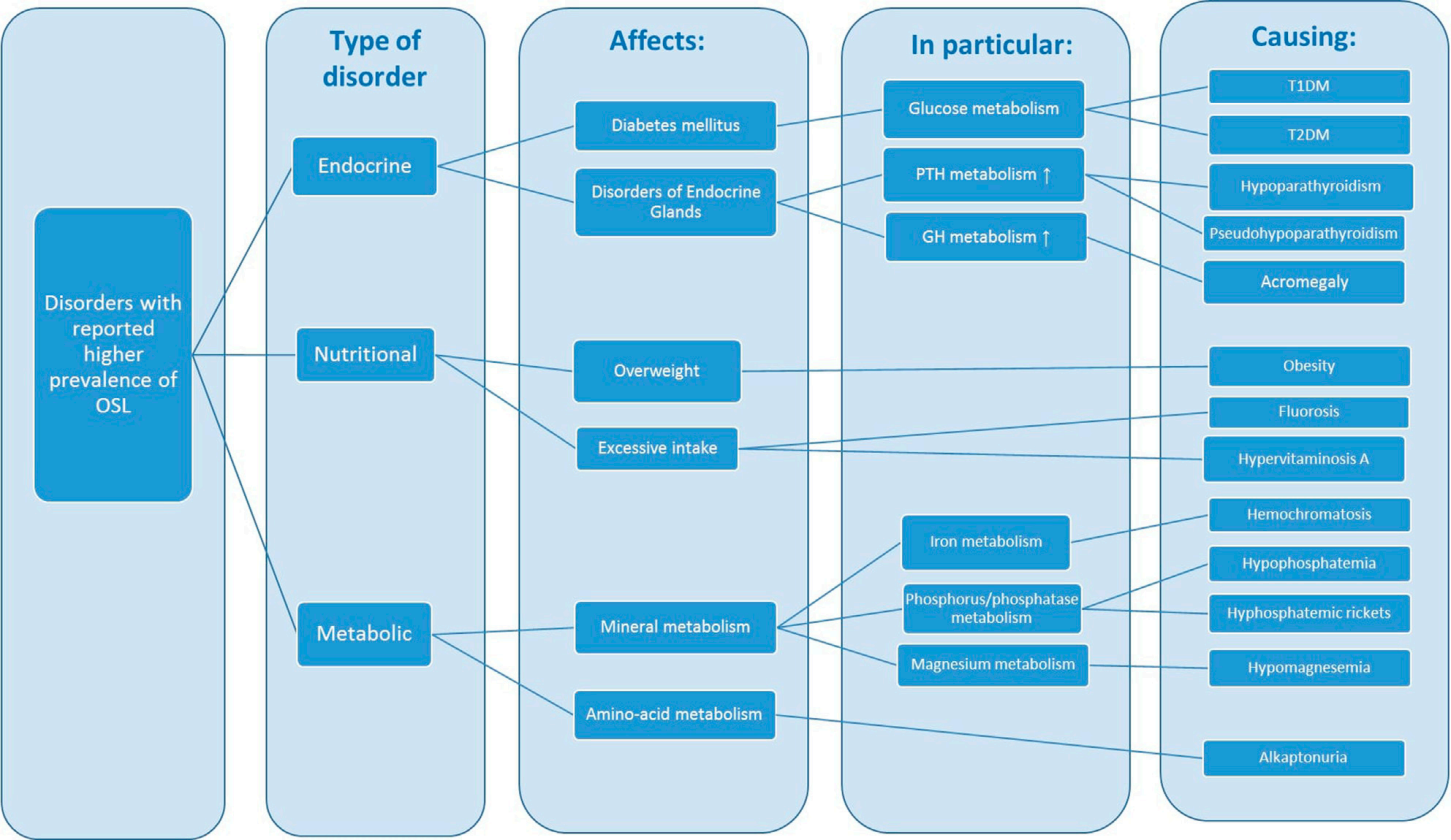
Disorders associated with a higher prevalence of OSL. GH: growth hormone, PTH: parathormone, IGF-A: insulin growth factor 1, HGA: homogentisic acid.

The presence of OSL has been described in association with numerous diseases of diverse etiologies. The type of disorders, the main pathways affected and the consequences, including the main anomalies identified in laboratory analysis, are outlined in Figure 1.

The OSL associated disorders can be of endocrine, nutritional or metabolic nature. The main endocrine associated disorders—diabetes mellitus, acromegaly and hypoparathyroidism—are characterized by disturbances in the metabolism of glucose, growth hormone (GH), and parathyroid hormone (PTH), leading to hypocalcemia, hyperphosphatemia, hyperglycemia and hyperinsulinemia. These endocrine anomalies are often linked to obesity, which can also have a strong genetic basis. The excessive intake of fluoride and vitamin A leads to OSL resembling DISH. Disturbances in mineral metabolism namely phosphorus phosphatase and calcium can also originate disorders that have been reported in association with OSL: familial hypocalciuric hypercalcemia, hypophosphatemic rickets and hypophosphatasia.

### 5.1 Monogenic disorders

Table 4 lists a subset of DISH and OPLL cases originated by monogenic disorders. With the exception of alkaptonuria, characterized by the levels of Homogentisic acid, all of the other disorders are directly involved in calcium and phosphate homeostasis. As expected, genes related in hypophosphatemic rickets and hypophosphatasia are directly involved in phosphate homeostasis. However, the reports of OSL are not related to all types of hypophosphatasia disorders. Saito et al. (2011), reported a case of OPLL with hypophosphatemic rickets/osteomalacia caused by a splice donor site mutation in the *ENPP1* gene. Cases of hypoparathyroidism associated with changes similar to DISH are also reported in the literature (Lambert and Becker, 1989; Unverdi et al., 2009; John and Suthar, 2016). The genes *GNAS*, *GCM2* and *PTH*, closely related to hypoparathryroidism, play a role in both calcium and phosphorus metabolism. According to what we know, there is only one case described of a patient with DISH and familial hypocalciuric hypercalcemia (FHH). The patient, a 45-year-old diabetic woman, have hypercalcemia secondary to FHH and developed dysphagia because of external esophageal compression from DISH. According to the authors, the relationship between FHH and DISH remains unproven (Rivas and Lado-Abeal, 2013). Acromegaly is a rare condition of high elevated somatic growth and distorted proportions arising from hypersecretion of growth hormone (GH) and insulin-like growth factor 1 (IGF-1) due to adenomas and pathogenic pituitary secretion (Ben-Shlomo and Melmed, 2008). According to Altomonte et al. (1992), GH levels may act as bone promoting factors in DISH.

**TABLE 4:** Monogenic disorders previously associated with OSL. Lack of inheritance means that it is still unconfirmed.

Disorder	Inheritance	OMIM	Gene/Locus involved
Hypophosphatemic rickets/osteomalacia	AD	193100	*FGF23*
	AR	241520	*DMP1*
	AR	613312	*ENPP1*
	AR	241530	*SLC34A3*
	XLD	307800	*PHEX*
	XLR	300554	*CLCN5*
Hypophosphatasia	AR	241500	*ALPL*
	AR	241510	
	AR, AD	146300	
Pseudohypoparathyroidism	AD	103580	*GNAS1*
Hypoparathyroidism	AD	146200	*GCM2*
	AD/AR	146200	*PTH*
Alkaptonuria	AR	203500	*HGD*
Acromegaly	Somatic/AD	102200	*AIP*
		102200	*GNAS1*
	X linked	300943	*GPR101*
	AD	610755	*CDKN1B*
	AD	131100	*MEN1*
	Somatic	174800	*GNAS*
	AD	160980	*PRKAR1A*
Familial Hypocalciuric Hypercalcemia	AD	145980	*CASR*
	AD	145981	*GNA11*
	AD	600740	*AP2S1*

AbbreviationsAD -Autosomal Dominant, AR- Autosomal Recessive, XLD, and XLR - X-linked Dominant and Recessive.

### 5.2 “Risk-factor” complex disorders

The etiology of “risk-factor” OSL disorders is complex, and determined by the interaction of inherited and environmental factors, such as age, smoking, alcohol consumption, diet and physical inactivity. These factors, as already know, effect type 2 diabetes mellitus (T2D) and obesity, two of the known risk factor for developing DISH. Even though heterogeneous, there are some monogenic forms of these OSL disorders; see Table 5 for more details. Diabetes mellitus is considered to be a heterogeneous group of disorders having as a main characteristic persistent hyperglycemia (Pillai and Littlejohn, 2014). Obesity is considered a complex and a multifactorial disease, however there are monogenic cases reported that are related to mutations in genes of the leptin/melanocortin system involved in food intake regulation (Huvenne et al., 2016). It is interesting to see that genetic variants in *LEPR* gene, as occurs in the ZFR murine model, can cause obesity, hypercholesterolemia, hyperinsulinemia, hyperlipidemia and also ossification of spinal ligaments, similar to human OPLL (Okano et al., 1997). Furthermore, there are studies reporting increased levels of serum leptin in female patients with OPLL (Ikeda et al., 2011) (Feng et al., 2018) as well as in DISH patients (Tenti et al., 2017). The osteogenic effects of leptin/leptin receptor (LepR) in conjunction with mechanical stress, on the ossification of the posterior ligament, through its interaction with osteogenic markers such as osteopontin, osteocalcin and *RUNX2*, were also recently shown (Chen et al., 2018). It is also pertinent to mention that *ENPP1* is a predisposition gene for both obesity and type 2 diabetes. The importance of leptin/*LEPR* in disorders such as DISH, with an important metabolic association, remain to be revealed.

**TABLE 5:** Complex disorders previously associated with OSL. AD stands for Autosomal Dominant, AR for Autosomal Recessive. Lack of inheritance means that it is not confirmed.

Disorder	Type	Inheritance	OMIM	Gene/Locus involved
Non-insulin-dependent Type 2 Diabetes mellitus	Monogenic - MODY	AD	606391	Genetically Heterogeneous—associated with mutations in 13 genes
	Polygenic		125853	Many susceptibility locus identified, including in *ENPP1*
Abdominal Obesity - Metabolic Syndrome	Monogenic	AR	615812	*DYRK1B*
			200100	*MTP*
	Polygenic		605552	*AOMS1*
				*AOMS2*
Obesity	Monogenic	AR	614962	*LEP*
		AR	614963	*LEPR*
		AR	600955	*PCSK1*
		AR	609734	*POMC*
	Polygenic		601665	Genetically heterogeneous but including *ENPP1* as susceptibility gene

### 5.3 Other rheumatic disorders coexisting with Ossification of Spinal Ligaments

The co-existence of DISH with other rheumatic disorders was first reported in 1950 by Forestier and Rotes Querol (Forrestier, 1950). Subsequent studies indicate, in some cases that up to 50% of DISH patients also have OPLL proposing that they share common etiopathogenic factors. Simultaneous OPLL and OLF are also very common in the literature (Li et al., 2012; Onishi et al., 2016). In addition, the co-existence of the three OSL disorders—DISH; OPLL and OLF has also been described in the literature (Guo et al., 2011). The association of DISH with psoriatic arthritis in the literature (Ben-Shlomo and Melmed, 2008) is common but studies concluded that is a side effect of retinoids treatment (Bologna et al., 1991). Other rheumatic diseases co-existing with DISH include: hyperostosis frontalis interna (Arlet et al., 1978; Mazières et al., 1978; Ciocci et al., 1985; Fukunishi et al., 1987; Fukunishi and Hosokawa, 1988), CPPD and/or CC (Resnick et al., 1978a; Bruges-Armas et al., 2006), gout (Resnick et al., 1978a; Littlejohn and Hall, 1982; Constantz, 1983; Fornaciari et al., 2009), rheumatoid arthritis (Resnick et al., 1978a; Resnick et al., 1978b; Forster et al., 1981; Mata et al., 1995), osteoarthritis (Resnick et al., 1978a), Heberden and Bouchard nodes (Schlapbach et al., 1992) and Paget’s disease (Mazières et al., 1978; Morales et al., 1993).

DISH and Ankylosing Spondylitis (AS) generally have a distinct radiographic appearance but sometimes, possibly in the early disease stages, they are difficult to distinguish radiologically (Williamson and Reginato, 1984; Olivieri et al., 1987; Olivieri et al., 1989; Rillo et al., 1989; Troise Rioda and Ferraccioli, 1990; Olivieri, 1991; Passiu et al., 1991; Maertens et al., 1992; Tishler and Yaron, 1992; Jattiot et al., 1995; Moreno et al., 1996; Kozanoglu et al., 2002; Jordana et al., 2009; Wooten, 2009; Macia-Villa et al., 2016; Kuperus et al., 2018). OPLL has also been observed in patients with AS but this coexistence is probably coincidental (Kim et al., 2007). Chondrocalcinosis, is characterized by the deposition of calcium containing crystals in synovial membranes, articular cartilage and, sometimes it can also affect periarticular soft tissues. Curiously, in some patients, the deposition of calcium crystals—hydroxyapatite or CPPD—can also occur in the spinal ligaments (Resnick and Pineda, 1984; Muthukumar et al., 2000) but this is usually difficult to differentiate from ossification (Ehara et al., 1998). *ANKH* is the only monogenic cause identified for CC (Table 6); a recent study described a gain-of-function mutation in the gene *TNFRSF11B*, which resulted in early-onset osteoarthritis and CC (Ramos et al., 2015). A recently described hereditary autosomal recessive ectopic mineralization syndrome in patients with arterial Calcification due to deficiency of CD73 (ACDC), was the result of a loss of function mutations in the 5′-nucleoside Ecto (*NT5E*) gene. These patients had erosive peripheral arthropathy and axial enthesopathic calcifications, resembling DISH although with decreased disc space height and the presence of large intervertebral disk calcifications (Cudrici et al., 2021). The similarities to both DISH and AS of the outcome of spine imaging of ACDC patients are noteworthy.

**TABLE 6:** Rheumatic disorders previously seen coexisting with OSL. AD stands for Autosomal Dominant, AR for Autosomal Recessive. Lack of inheritance means that it is not confirmed.

Disorder	Inheritance	OMIM	Gene/Locus involved	Mechanism
ACDC	AR	211800	NT5E	Pyrophosphate metabolism
Ankylosing Spondylitis	Multifactorial	106300	*HLA-B*	MHC Peptide presentation
	AD	183840	*SPDA2 locus*	
		613238	*SPDA3 locus*	
Chondrocalcinosis	AD	118600	*ANKH*	Pyrophosphate metabolism
	AD	600668	*CCAL1 locus*	
	AR	602643	*TNFRSB1*	

## 6 Discussion

### 6.1 Familial aggregation reports

The existence of a small number of family reports, with early-onset and exuberant phenotypes, in which the genetic cause was not identified and most of the times was not even investigated, raises the possibility that there are some cases of monogenic DISH and OPLL. There are possibly three main types of OSL: A sporadic form, a type that is secondary to associated metabolic disorders and a hereditary type. It is now clear that most OSL cases do not follow a simple, single gene Mendelian inheritance pattern, but instead are multifactorial disorders developing in individuals with a genetic predisposition from a variety of genetic variants in different genes.

### 6.2 Animal models

The existence of spontaneous and manipulated animal models for both DISH and OPLL could facilitate the identification of causal human genetic factors. It seems probable that the human phenotype of OPLL and DISH are likely to be caused by mutations in genes that underlie the animal models for these disorders. As far as we know, there are no reports of *SLC29A1* (ENT1 mice model for DISH) human gene mutations in association with DISH. The association of *ENPP1* with OPLL susceptibility (31, 47–49) is still unsubstantiated (50). Interestingly, in one study the authors found that the combination of variants in *ENPP1* and *LEPR* genes was associated with the location and extension of OPLL (51). An interesting report about hypophosphatemic rickets in an OPLL patient due to a homozygous mutation in the *ENPP1* gene (53), substantiates the likely importance of this gene in the etiopathogenic mechanism of OSL.

The case of the *ank* mouse has been quite different. In humans, analysis in the *ANKH* gene has identified several mutations that segregate with CC phenotype but only in a very limited subset of pedigrees. The co-coexistence of spinal ossification with CC is well supported in the literature (10, 12, 13), indicating a strong genetic link between these disorders. The genetic confirmation between spinal ossification and CC comes from two animal models—twy and *ankh* mice—the mouse models for OPLL and CC, develop spinal ossification and hydroxyapatite arthropathy. Both genes, *ENPP1* and *ANKH*, regulate PPi levels thus having an essential role in bone mineralization and soft tissue calcification. The association of *ENPP1* variants with Chondrocalcinosis, is considered a minor determinant of the disease (58, 59).

### 6.3 Genetic variants association

Three different genetic variants in *COL6A1* have been associated with both DISH and OPLL. Results from these studies are inconsistent due to the type of variant associated, the lack of explanation of the pathogenic mechanism and the low numbers of individuals studied. Further progress in investigation of DISH requires a concerted approach, similar to the ones used to target the genetic basis of OPLL. In the latter case linkage studies, candidate gene association studies and even genome wide association studies were performed and revealed that OPLL is genetically heterogenous. Despite all the studies, and the large number of genes that have been associated with OSL, most of the associations are still inconsistent because genetic variants were localized in non-coding regions. Several genes involved many potential low risk effects in OSL inheritance, so there is insufficient power and analysis for their detection.

### 6.4 Genetics of associated disorders

The higher prevalence of OSL in patients with endocrine, nutritional and metabolic disorders made us wonder if the known genetic cause for these associated disorders could help to clarify the putative genetic pathways involved in the etiology of OSL. The ectopic calcification occurring is most probably predisposed by the balance between the expression of specific genes that act directly or indirectly on the phosphorus to calcium ratio. The crucial role of angiogenesis in DISH etiology has also been suggested, as it might be the common pathogenic background of some conditions included in metabolic syndrome. Nonetheless, there are several case reports of patients with monogenic metabolic disorders with the occurrence of DISH and OPLL.

## 7 Conclusion

A validated set of classification criteria for diseases characterized by ectopic mineralization of spinal tissues is of utmost importance for genetic studies so homogeneous phenotype groups can be established for investigation. This is particularly important in DISH because this disease is characterized by the ossification of the anterior spinal ligaments and generalized symmetrical enthesopathic calcifications, which may well be among the first manifestations of the disease or the main evidence of the disease in a subset of patients. At this time, DISH disease is requiring a validated set of criteria to robustly describe and establish homogeneous cohorts of patients. A more comprehensive designation of DISH, including patients with early phase disease, are clearly indispensable for genetic studies (Mader et al., 2013). On the other hand, great advances have been made in understanding the presentation of different types of OPLL.

Taken together the collected evidence suggests OSL has a heterogeneous genetic basis. The rapid advance in methods for genetic studies has brought new and interesting insights into ectopic calcification, and is providing confirmation about the importance of genes for the regulation of Pi/PPi levels, which control mineralization. Future genome-scale approaches will contribute to pinpoint susceptibility genes. However, to provide sufficient analytical power, the number of patients needs to be enlarged and the clinical/radiological disease classification, especially in DISH patients, needs substantial improvement. International collaborations are essential to increase sample size and overcome analytical challenges caused by the genetic heterogeneity of these complex diseases of calcification.
