# Corrigendum: Evidence for a genetic contribution to the ossification of spinal ligaments in ossification of posterior longitudinal ligament and diffuse idiopathic skeletal hyperostosis: a narrative review

**DOI:** 10.3389/fgene.2024.1477788

**Published:** 2024-08-30

**Authors:** Ana Rita Couto, Bruna Parreira, Deborah M. Power, Luís Pinheiro, João Madruga Dias, Irina Novofastovski, Iris Eshed, Piercarlo Sarzi-Puttini, Nicola Pappone, Fabiola Atzeni, Jorrit-Jan Verlaan, Jonneke Kuperus, Amir Bieber, Pasquale Ambrosino, David Kiefer, Muhammad Asim Khan, Reuven Mader, Xenofon Baraliakos, Jácome Bruges-Armas

**Affiliations:** ^1^ Hospital de Santo Espirito da Ilha Terceira EPER, SEEBMO, Angra do Heroísmo, Portugal; ^2^ Comprehensive Health Research Centre, Hospital de Santo Espírito da Ilha Terceira, Lisbon, Portugal; ^3^ University of Algarve, Center of Marine Science (CCMAR), Faro, Portugal; ^4^ Hospital de Santo Espirito da Ilha Terceira EPER, Orthopedics Service, Angra do Heroísmo, Portugal; ^5^ Centro Hospitalar Do Medio Tejo EPE Unidade de Torres Novas, Rheumatology Department, Santarém, Portugal; ^6^ CHRC Campus Nova Medical School, EpiDoc Research Unit, CEDOC, Lisboa, Portugal; ^7^ Emek Medical Center, Rheumatology Unit, Afula, Israel; ^8^ Sheba Medical Center, Tel Aviv, Israel; ^9^ Luigi Sacco University Hospital, Rheumatology Unit, Milano, Italy; ^10^ Istituti Clinici Scientifici Maugeri IRCCS, Neuromotor Rehabilitation Unit of Telese Terme Institute, Pavia, Italy; ^11^ Universita Degli Studi di Messina, Rheumatology Unit, Clinical and Experimental Medicine, Messina, Italy; ^12^ University Medical Centre, Department of Orthopedics, Utrecht, Netherlands; ^13^ Universitair Medisch Centrum Utrecht, Utrecht, Netherlands; ^14^ Istituti Clinici Scientifici Maugeri IRCCS, Cardiac Rehabilitation Unit of Telese Terme Institute, Pavia, Italy; ^15^ Ruhr-Universitat Bochum, Rheumazentrum Ruhrgebiet, Bochum, Germany; ^16^ Case Western Reserve University, Cleveland, OH, United States; ^17^ Rappaport Faculty of Medicine, Technion, Haifa, Israel; ^18^ Ruhr University Bochum, Rheumazentrum Ruhrgebiet, Herne, Germany

**Keywords:** ossification, genetics, ectopic calcification, diffuse idiopathic skeletal hyperostosis, ossification of posterior longitudinal ligament

In the published article, there was an error in **Affiliation** number 2. Instead of “CHRC Campus Nova Medical School, Lisboa, Portugal”, it should be “Comprehensive Health Research Centre, Hospital de Santo Espírito da Ilha Terceira, Lisbon, Portugal.”

In addition, there was an error in the **Funding** statement. The correct **Funding** statement is “The present publication was funded by Fundação Ciência e Tecnologia, IP national support through CHRC (UIDP/04923/2020).”

The authors apologize for these errors and state that this does not change the scientific conclusions of the article in any way. The original article has been updated.

